# The interplay of RALF structural and signaling functions in plant-microbe interactions

**DOI:** 10.1371/journal.ppat.1013588

**Published:** 2025-10-15

**Authors:** Sebastian Schade, Henriette Leicher, Michelle von Arx, Isabel Monte, Julien Gronnier, Martin Stegmann

**Affiliations:** 1 Molecular Botany, Institute of Botany, Ulm University, Ulm, Germany; 2 Chair of Phytopathology, TUM School of Life Sciences, Technical University of Munich, Freising, Germany; 3 Plant Cell Biology, TUM School of Life Sciences, Technical University of Munich, Freising, Germany; 4 Center for Plant Molecular Biology (ZMBP), University of Tuebingen, Tuebingen, Germany; University of Tübingen: Eberhard Karls Universitat Tubingen, GERMANY

## Abstract

Rapid alkalinization factor (RALF) peptides are important signaling molecules binding to Catharanthus roseus receptor-like kinase 1-like (CrRLK1L)—LORELEI/LORELEI-like GPI-anchored protein complexes to regulate a plethora of physiological responses, including plant-microbe interactions. Recently, RALF peptides were demonstrated to possess additional structural functions as organizers of primary cell wall structure through binding to de-methylated pectin via Leucine-rich repeat extensin proteins. In this review, we discuss these novel findings and their possible implications for RALF-regulated plant-microbe interactions, put them into context with the well-known signaling function of RALF-CrRLK1L complexes, and address key future directions for this emerging field in molecular stress physiology and beyond.

## Introduction

Rapid alkalinization factor (RALF) peptides are central regulators of plant physiology modulating diverse aspects of growth, development, and reproduction [1–10]. As a molecular basis explaining their function, RALFs were initially solely described as ligands for plasma membrane (PM) localized receptor complexes consisting of Catharanthus roseus receptor-like kinases 1-like (CrRLK1Ls) and GPI-anchored proteins of the LORELEI/LORELEI-like GPI-anchored protein (LRE/LLG) family. FERONIA (FER) and LLG1 play a predominant role in RALF perception and are expressed in most tissues. Their downstream responses and pathway components are best characterized among CrRLK1L modules [11–14]. Upon RALF perception, FER initiates phosphorylation of several downstream executors. Among others, this includes FER-dependent phosphorylation of Arabidopsis H+ATPase 1 (AHA1)/AHA2 for pH homeostasis, as well as proteins involved in apoplastic ROS production, translation, splicing, and hormonal signaling [15].

Recently, RALF peptides were demonstrated to possess structural roles for tip-growing cells. Indeed, RALFs bind to cell wall (CW) located receptors, the Leucine-rich repeat extensin (LRX) proteins, and to pectin chains, forming ring-shaped and meshwork-like supramolecular structures that support cell wall integrity [2,9], hence highlighting dual structural and signaling roles of individual RALF peptides. RALF peptides display high affinity to LRX proteins, predominantly at acidic conditions. Binding to FER-LLG complexes are a order of magnitude weaker and supported by alkaline pH [14,16]. This raises the question how both modules cooperate for physiologically optimal RALF binding. In addition, RALFs are central regulators of plant-microbe interactions by, e.g., regulating immune receptor complexes and PM dynamics, as well as defense-related phytohormone signaling [12,17,18]. This function is to date primarily linked to their role as CrRLK1L-LLG-dependent signaling molecules. The exciting discovery of RALFs as CW components now raises important questions about the interplay between RALF structural and signaling roles. In this review, we will discuss emerging questions about these findings with a focus on plant-microbe interactions.

## Question 1: What is the interplay of RALF cell wall binding and receptor-dependent signaling?

The C-terminus of RALFs binds to LRXs to expose positively charged cationic residues that engage in physical interaction with de-methylated and negatively charged pectin and constitute a structural element of the CW [2,9]. Since, the affinity of RALF peptides for LRX is of an order of magnitude higher than for its PM-localized receptors, it is hypothesized, but not experimentally validated, that RALFs are released during CW remodeling (e.g., during cell expansion) and may promote CrRLK1L-dependent cell wall signaling (CWS) to induce compensatory responses.

**Fig 1:**
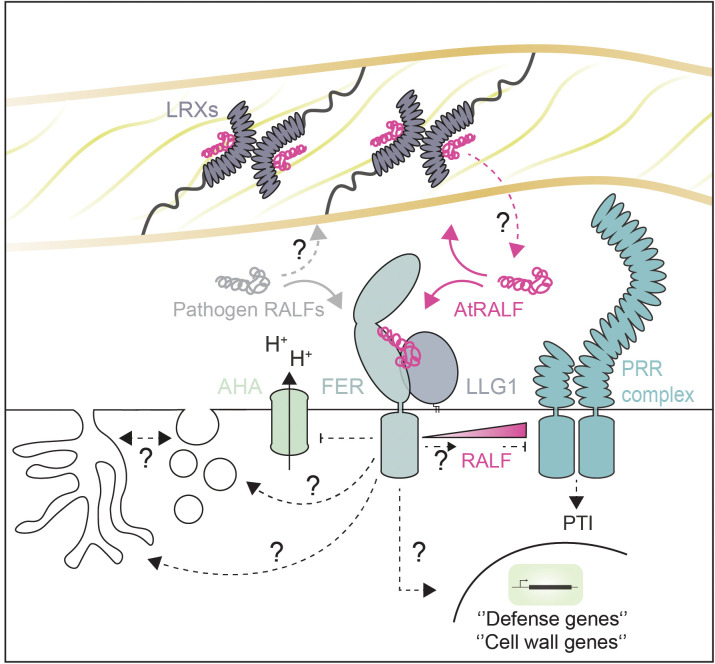
Endogenous rapid alkalinization factors (RALFs) bind to wall-located leucine-rich repeat extensins (LRXs) and/or plasma membrane (PM) localized receptor complexes, e.g., FER-LLG1. RALF binding to FER-LLG1 negatively regulates FLS2-BAK1 complex formation at high concentration, but promotes pattern recognition receptor complex formation at low concentration. Perception of RALFs by FER-LLG1 induces apoplast alkalinization, which modulates cell wall (CW) status by regulating the activity of pectin-modifying enzymes, which in turn could modulate RALF-pectin binding sites. Further, alkalinization can modulate the affinity of RALF peptide to their CW and PM receptors. The availability of CW-located RALF binding sites has been proposed to define the balance of RALFs perceived by LRXs or FER-LLG1. Thereby, RALF perception by FER-LLG1 could also be informative of the CW status and initiates adaptive responses such as the expression of CW-related genes alongside immunity-related genes. CW perturbations during cell expansion, as well as microbe infection, have been hypothesized to release CW-located RALFs and thereby modulate FER-LLG1-dependent immune and CW signaling. RALFs induce FER-LLG1 endocytosis as well as large-scale PM invaginations, which could serve for microbial invasion and/or the establishment of feeding structures. However, whether and how RALF signaling-dependent endocytosis and large-scale invaginations are connected remains unclear. Pathogen-derived RALFs hijack FER-LLG1-dependent signaling. However, whether they also manipulate CW sensing/signaling and remodeling by the RALF-LRX module or via an unknown process remains unclear.

A hallmark of RALF-induced signaling is apoplastic alkalinisation, which directly affects the activity of pectin methylesterases (PMEs), enzymes determining CW pectin methylation status and hence the availability of CW RALF binding sites [19,20]. This suggests a tight interplay and mutual dependence of both RALF roles. Consistently, *fer* mutants display disrupted apoplastic pH homeostasis and altered PME activity [11,21–23]. In addition, FER is associated with sensing perturbations in CW mechanics, potentially inducing gene expression related to CW remodeling [24–27] (Fig 1). RALF-mediated CWS is required to maintain cellular anisotropy in an interplay with brassinosteroid (BR) signaling, a growth-regulating hormone [22]. Further, BR signaling promotes the PM-localization of FER, thereby safeguarding cell expansion [28]. LRX mutants also display altered PME activity [21]. This may be caused by a yet unknown sensing mechanism for LRX-RALF devoid CWs, which causes feedback signaling to boost PME activity. It is not clear whether genetic disruption of LRX is a prerequisite for this observation or may also occur during regular cell elongation or growth. However, it may also be related to impaired RALF secretion and PM signaling for pH adjustments by FER-LLG1 complexes, as illustrated by the impairment of RALF22 secretion in root hairs of *lrx1 lrx2* mutants [2]. It will be important to reveal how LRX-RALF and FER-LLG1-RALF modules cooperate to modulate CW status and compensatory responses. Several studies point towards a direct monitoring of CW status by FER [25,27], but whether FER directly senses a wall-derived ligand or detects changes in CW mechanics remains unknown. Also, the large diversity of RALFs, with at least 37 members in Arabidopsis [29], offers the possibility of charge-dependent differential functions linking CW status to PM signaling.

## Question 2: What are the roles of RALF peptides in immunity?

RALF-mediated regulation of immunity has been primarily linked to their signaling function and perception by CrRLK1Ls-LLGs. For instance, RALF23, a bona fide ligand for FER-LLG1 complexes, regulates the formation of elicitor-induced pattern recognition receptor (PRR) complexes and PRR-triggered immunity (PTI) signaling in a concentration-dependent manner. At high concentration, RALF23 inhibits flagellin-induced Flagellin Sensing 2 (FLS2)-Brassinosteroid Insensitive-Associated Kinase 1 (BAK1) complex formation [12,17,30,31]. The inhibitory effect of RALF23 relies on FER kinase activity, occurs rapidly, and has been linked to the formation of PM-associated RALF-pectin condensates, and to the regulation of FLS2 and BAK1 PM nano-organization [32]. At low nanomolar concentrations, RALF23 promotes FLS2-BAK1 complex formation via an unknown mechanism [30]. Interestingly, LRXs are also genetically required for ligand-induced FLS2-BAK1 complex formation and PTI signaling [17], suggesting that both structural and signaling roles underlie RALFs function in PTI. Changes in CW chemistry and/or CW damages that occur during pathogen infection may release RALF peptides and, by this, fine-tune immune responses. Lower free RALF concentrations may promote resistance, while CW rupture may inhibit immunity to prioritize RALF-dependent tissue repair [12,30,33]. Accordingly, the dose-dependent role of RALFs may represent a regulatory continuum responsive to the infection stage or the identity of the invading microbe (Fig 1).

RALF-FER downstream signaling further regulates stability of the transcription factor MYC2 to modulate jasmonic acid levels [18] and proteolytic cleavage of the FER kinase domain, enabling nuclear translocation and activation of root localized immunity [30]. In roots, the RALF23-FER module is integrated in low-phosphate responses, inhibiting PRR complex formation and facilitating beneficial microbial association [31,34]. Whether RALF-pectin interaction is involved in these outputs remains to be clarified. Other CrRLK1Ls have also been implicated in immunity. ANXUR1 inhibits FLS2-BAK1 complex formation, while LETUM1 (LET1) and LET2 associate with LLG1 to control autoimmunity and activation of a Nucleotide Binding Leucine-Rich Repeat intracellular immune receptor [35–37]. Whether this is mediated by RALF perception and CW status remains unknown.

## Question 3: Do some microbes require/use RALF-mediated PM invaginations to invade cells/tissues?

The perception of RALF1 and RALF23 at the cell surface induces important cell membrane reorganization. First, RALF-pectin oligomers form PM-associated molecular condensates, which nucleates FER-LLG1 complex formation, triggers the PM reorganization and the endocytosis of non-cognate PM proteins, such as FLS2, BRI1, and BAK1 [32]. Surprisingly, fluorescently tagged RALF1 induced endocytosis but remained CW localized, raising the question whether RALF release is required for receptor perception or whether LRX-dependent CW associations are a prerequisite for RALF signaling functions [32]. RALF1 and RALF23 also induce FER-dependent and LRX-independent PM invaginations in root cells, which was inhibited by interfering with PME activity [22,38]. Several microbes rely on PM invagination for host association [39], which raises the question whether RALFs can promote intracellular microbe accommodation. Symbiotic rhizobia rely on PM invagination for the formation of infection threads [40]. During nodule establishment, de novo invagination of the basal membrane at the passage site of the infection thread to neighboring cells is a critical step for successful colonization and regulated by *Medicago truncatula* (Mt) RALF1 [39,41,42]. Conversely, knockdown of MtFER reduces nodulation factor responses and infection thread development, diminishing rhizobial colonization, but also root hair formation, making symbiosis-specific interpretations difficult [43]. Several plant-associated microbes and nematodes produce RALF-like peptides, too [44–46] (Fig 1). The RALF-encoding beneficial endophytic ascomycete *Colletotrichum tofieldiae* (*Cto*) [47] invades Arabidopsis roots through epidermal junctions or directly into epidermal cells to form intercellular hyphae. PM marker studies indicate localized invagination around these hyphae, likely facilitating biotrophic interaction [48]. Interestingly, colonization efficiency is significantly reduced in both *fer* mutant plants and *Cto* ΔCtRALF, suggesting that CtRALF-FER complex formation is critical for successful root accommodation, but whether it directly affects intracellular host establishment remains unknown [47]. Future research should elucidate the role of RALFs for cellular microbe establishment by PM invaginations, a process that might be dependent on their role as CW-PM interface molecules.

## Question 4: Do pathogen RALF mimics show sequence-specific functional specialization to support colonization?

Functional characterization of RALFs from the fungal pathogen *Fusarium oxysporum* (*Fox*) (F-RALFs) and the nematode *Meloidogyne incognita* (MiRALFs) revealed that F-RALFs and MiRALFs trigger canonical RALF responses *in planta* and promote infection and parasitism, respectively. The effect of F-RALFs and MiRALFs depends on *FER*, consistent with the conservation of the YISY motif that facilitates LLG binding and LLG-FER complex formation [14,44,46]. However, it appears that some fungal and nematode RALFs show striking differences to plant RALFs*.* RALFs contain four conserved C residues that establish disulfide bonds in folded RALFs, but nematode RALFs lack the first C pair [46]. Conversely, nematode RALFs and most fungal RALFs lack a critical Y pair corresponding to AtRALF4^Y83Y84^ required for LRX association, with the exception of *Macrophomina phaseolina* and *Botryosphaeria dothidea* RALFs [45]. *Fox* mainly invades through natural openings and wounds and grows in the plant apoplast before entering the xylem. Cell invasion is the exception and associated with the loss of plant cellular integrity [49]. Root knot nematodes migrate intercellularly through the root before entering the vasculature, where they induce the formation of feeding structures that require stylet-mediated cell penetration. This raises the question whether immuno-suppressive RALFs without predicted CW binding properties may be advantageous for specific pathogen lifestyles. Given the conservation of other amino acids in the same position in plant and nematode RALFs (i.e., PN/PV/PY), it is still possible that these residues mediate binding to LRXs through an alternative mechanism, and it remains unknown whether their function is independent of LRXs. Conversely, the net charge of MiRALFs and fungal RALFs is comparable to plant RALFs, suggesting that the polycationic nature of all these RALFs could also mediate binding to the CW. In addition, *Cto* RALFs lack the conserved YY motif, but *Cto* likely requires CW perturbation for cell invasion [47].

Collectively, sequence differences of several RALFs from plant-associated organisms raise the question whether they manipulate immunity or reprogram host physiology independent of CW structural perturbations or may rely on alternative mechanisms for executing this function. This may be part of elaborate infection strategies, the mechanistic nature of which requires future investigation. However, plant CW modification is likely relevant for *M. incognita* to induce the formation of host cell-derived feeding structures [50]. MiRALFs may contribute to this process by manipulating cell expansion and CW remodeling, which may involve the targeting of both CW and PM RALF receptors. An important future research subject is to understand whether and how F-RALFs and MiRALFs do rely on RALF-mediated CW organization for infection, whether their sequence-divergent RALFs do affect plant physiology mechanistically different from analyzed plant LRX-binding RALFs, and what may be the underlying mechanism.

## Question 5: What is the biological relevance of RALF-mediated plant modulation for pathogen fitness beyond immune suppression and CW remodeling?

Immune suppression and PM invagination are likely not the only virulence functions of RALFs, and they do not appear to affect microbial intracellular accommodation in some plant-microbe interactions. Powdery mildew fungi penetrate into adaxial leaf epidermal cells and do not produce RALF mimics. Their infection success is diminished in both *fer* and *ralf* mutants, suggesting that they rely on plant endogenous RALF function (Fig 1), but neither penetration nor hyphal formation was compromised in those mutants [21,51]. RALFs are potent inducers of apoplastic alkalinization—a response particularly important during infection by hemibiotrophic fungi [52]. In *Fox*, loss of RALF-like genes reduces the ability to induce plant alkalinization to activate the mitogen-activated protein kinase Fmk1 for successful infection [44]. Increasing the external pH to 7 can partially rescue the virulence of RALF-deficient *Fox* strains [44]. Interestingly, initial *Fox* hyphal contact with roots induces acidification, highlighting the necessity of dynamic pH modulations for infection success [53]. Similarly, powdery mildew infection relies on dynamic apoplastic pH conditions [21]. Changes in pH may affect nutrient availability and symporter/antiporter transport processes for successful pathogen feeding post-host establishment [54], as illustrated by unaffected cell invasion but reduced reproductive success of powdery mildew fungi on FER/RALF mutants or upon apoplastic pH disturbance. Moreover, RALF1-FER regulates protein translation and target of rapamycin activity in roots and root hairs [4,5], but whether this is beneficial for root-infecting pathogens and induced by pathogen RALF mimics requires further investigation.

## Conclusions

The complexity of the interplay between RALF CW binding and receptor-dependent signaling functions is only beginning to emerge and has potential profound implications on our understanding how these versatile peptide hormones/CW binding blocks confer adaptability during plant-microbe interactions and beyond. To address the challenging questions discussed in this review, genetic studies with targeted RALF mutations in both plant and microbial RALFs are required, as well as modeling and structural approaches to understand diverse RALF module surface and charge properties underlying distinct roles. Moreover, advanced cell biological methods are required to study dynamic CW binding and release of RALFs to complement their dual function, as well as detailed understanding of spatiotemporal secretion of sequence-divergent microbial RALF mimics that may support distinct pathogen lifestyles.
